# Same-sex sexual behavior in brown-headed spider monkeys (*Ateles fusciceps fusciceps*) during grappling between two subadult males

**DOI:** 10.1007/s10329-024-01147-3

**Published:** 2024-08-07

**Authors:** Malika Gottstein, Citlalli Morelos-Juárez, Colleen M. Schaffner, Filippo Aureli

**Affiliations:** 1 Eva Mayr-Stihl Professorship for Forest Genetics, Faculty of Environment and Natural Resources, Albert-Ludwigs-Universität Freiburg, Freiburg im Breisgau, Germany; 2 Fundación Reserva Tesoro Escondido, Quito, Ecuador; 3 Psychology Department, School of Humanities and Social Sciences, Adams State University, Alamosa, CO USA; 4 Instituto de Neuroetología, Universidad Veracruzana, Xalapa, Mexico; 5 Research Centre in Evolutionary Anthropology and Palaeoecology, Liverpool John Moores University, Liverpool, UK

**Keywords:** *Ateles fusciceps*, Brown-headed spider monkey, Grappling, Same-sex sexual behavior

## Abstract

Sexual behavior in animals fulfills reproductive and social functions, extending beyond the traditional focus on reproduction. Same-sex sexual behavior, defined as genital contact or manipulation between individuals of the same sex, occurs in various primate species. In spider monkeys, grappling, a behavior involving prolonged mutual embraces, face greeting, tail intertwining, and genital manipulation, occurs primarily between males. Here, we report a novel incidence of same-sex sexual behavior and grappling between two subadult male brown-headed spider monkeys (*Ateles fusciceps fusciceps*). Our observation contributes to the understanding of the social functions of sexual behavior and to the broader appreciation of primate sexuality.

## Introduction

The primary function of sexual behavior is reproduction, but a broader view encompasses a spectrum of functions beyond reproduction (Bagemihl 1999; Sommer and Vasey 2006). This broader view includes same-sex sexual behavior (SSB), defined as genital contact or genital manipulation between individuals of the same sex (Vasey 1995; Delval et al. 2023). In primates, SSB is common in many species, predominantly in Catarrhini (e.g., baboons (Smuts and Watanabe 1990), bonobos (Moscovice et al. 2019)), with occasional reports in Platyrrhini (Vasey 1995; Dixson 2010; Schaffner et al. 2012; Busia et al. 2018; Rufo and Ottoni 2020; França et al. 2023; Delval et al. 2023). The functions of SSB in primates are diverse and include facilitating communication and coordination, forming alliances, asserting dominance, supporting reconciliation and regulating tension (Smuts and Watanabe 1990; Vasey 1995; Busia et al. 2018; Delval et al. 2023). The function of SSB can vary significantly between species (e.g., due to differences in social organization and environmental context) and within species (e.g., influenced by sex, age classes, and dominance rank). Historically, reports of SSB in primates were overlooked or dismissed, due to normative concepts and restrictions on human sexual behavior (Vasey 1995). Recognizing the importance of non-reproductive sexual behaviors, including SSB, allows for a more accurate understanding of primate behavior.

Spider monkeys live in multi-male, multi-female groups with a high degree of fission–fusion dynamics, in which individuals split and merge into subgroups with variable composition throughout the day (Aureli and Schaffner 2008). Males, being the philopatric sex, develop strong cooperative relationships with one another (Schaffner et al. 2012; Saldaña-Sánchez et al. 2022). Although spider monkey males exchange more affiliative interactions, such as grooming, with other males than with females, they also exchange more embraces, which serve to reduce the likelihood of aggression and uncertainty in circumstances such as subgroup fusions and interactions with mothers of young infants (Aureli and Schaffner 2007; Slater et al. 2007, 2009). Spider monkeys are not considered to be seasonal breeders but there seem to be birth peaks correlated with fruit availability (Campbell and Gibson 2008).

Spider monkeys engage in a complex social behavior-labeled grappling, consisting of a prolonged series of mutual embraces, face greeting and touching, intertwining of tails, and mutual or unidirectional manipulation of genitalia with mouth, hands or feet (Eisenberg and Kuehn 1966; Schaffner et al. 2012; Busia et al. 2018). Although grappling was initially described as a male–female interaction (Eisenberg and Kuehn 1966), it is predominantly observed in male-male dyads, usually initiated by younger males toward older males (Aureli and Schaffner 2008; Schaffner et al. 2012). Grappling appears to regulate male social relationships, and is frequently observed when younger males seek affiliative interactions with older males (Slater et al. 2009; Schaffner et al. 2012).

Grappling meets the criterion for sexual behavior when genital manipulation occurs between the partners. In Geoffroy’s spider monkeys (*A. geoffroyi*), instances of SSB during grappling have been documented (Busia et al. 2018).

Here, we present an observation of SSB between two subadult male brown-headed spider monkeys (*A.* *fusciceps fusciceps*) within a grappling bout with mutual genital manipulation. Given the inherent challenges in observing spider monkey sexual behavior (Campbell and Gibson 2008), our observation contributes to the limited body of behavioral observations on this species.

## Methods

### Study species and site

*Ateles fusciceps* is distributed in the Chocó ecoregion, covering south-eastern Panama, Colombia and Ecuador west of the Andes. The species is classified as “endangered” by the IUCN and has been listed as one of the 25 most endangered primates worldwide (Moscoso et al. 2020; Tirira et al. 2022). The subspecies *A. f.* *fusciceps* is endemic to north-western Ecuador. It is reduced to a population size of a few hundred individuals and listed as “critically endangered” in Ecuador (Tirira 2007, 2011, 2021; Moscoso 2010; Cervera and Griffith 2016).

We followed subgroups of this subspecies within the Tesoro Escondido reserve (0°28 ‘41 ‘‘—0°34 ‘19 ‘‘ N, 79°01 ‘44 ‘‘—79°11 ‘25 ‘‘ W), a private conservation area in the province of Esmeraldas, north-western Ecuador (see Morelos-Juárez et al. 2015 for details on the study area). The spider monkeys at the study site are not fully habituated. When we meet them, at least one monkey usually reacts to our appearance by rustling branches, coming down from the canopy and calling. After a few minutes, however, such behavior stops, and we can follow them. During the study period between March and August 2023, we followed subgroups for up to 7 h per day (mean contact time: 1h24min) for a total of 149 h.

Since we cannot distinguish between individuals, we do not know the exact number of spider monkeys at our study site. It is likely that there are three groups, separated by a river. When we follow the monkeys, we collect data on subgroup composition based on an estimation of the age class of each subgroup member. We consider individuals as adults when they are fully grown, subadults when they almost reach the size of an average adult and are moving independently, juveniles when smaller than subadults and mostly traveling with their mother, and infants when mostly carried by the mother. We distinguish females from males by the presence of a hypertrophied pendulous clitoris, characteristic for female spider monkeys (Campbell and Gibson 2008).

### Observation protocol

To search for subgroups of spider monkeys, MG and a parabiologist (trained local research assistant) started walking into the forest every morning between 7:00 and 9:00, alternating trails that cover the study area. Upon encountering a subgroup, we estimated its composition and followed it as long as possible while using behavior sampling to collect data on feeding (Bateson and Martin 2021). We noted social behavior ad libitum. We conducted observations using Leica Ultravid 10 × 42 binoculars.

When noticing the grappling, we first recorded the exact time and position of the involved individuals (GPS location, height in the tree and estimated distance to the rest of the group). Both observers carefully checked the sex and age class of the individuals involved at the beginning of the observation. During the grappling bout, we continually took notes on the observed behaviors, and their duration. We filmed short parts (121 s divided in two videos) of the grappling bout with a Samsung Galaxy A52 smartphone through binoculars, but the resulting footage is of poor quality. The observation described here is the only case of sexual behavior we observed during the 6 month study.

## Results

On 04/04/2023, MG and parabiologist JPE followed a subgroup from 08:50 to 12:32. The subgroup consisted of eight individuals: four adult females, one juvenile female and three subadult males. From 09:57 to 10:34, we observed a grappling bout between two of the subadult males. During this time, the two subadult males were at approximately 30 m from the rest of the subgroup, in the lowest branch of a tree, at a height of about 15 m.

Our attention was drawn to the two subadult males when we heard an ook-ook sound, which is associated with grappling (Eisenberg and Kuehn 1966; Busia et al. 2018). When we arrived at their location at 09:57, the two males were engaged in a ventroventral embrace while hanging from their prehensile tails. In the following minutes, the monkeys repeatedly wrapped their arms and legs around each other’s bodies, touched each other’s faces and upper bodies using hands, feet and mouth, while staying in close contact. Every few minutes, they interrupted the contact and sat on the branch in proximity, partly with intertwined tails. Then, they restarted the ventroventral embrace hanging from their prehensile tails below the branch. At 10:20, both males had erect penises, and we observed frequent mutual manipulation of genitalia using hands and feet, as well as rubbing their anogenital regions together. In addition, one male had repeated oral contact with the genitalia of the other male, but we could not determine whether he had the penis or testicles in his mouth. The oral contact was short (a few seconds) and interrupted. At 10:31, we noticed ejaculate on one males’ penis. We cannot say for sure what caused the ejaculation. During the grappling observation, we heard the other subgroup members whinny vocalizations several times. However, the subadult males did not respond to the calls until the end of grappling at 10:34, when they replied with a whinny and moved to join the other subgroup members.

## Discussion

Our observation is the first of SSB reported between two subadult male brown-headed spider monkeys. A wide range of types of sexual behavior have been observed in primates, encompassing courtship displays, manual-genital, oral-genital, and genital-genital stimulations, as well as mounting between different-sex and same-sex partners (Bagemihl 1999). The genital contact and manipulation we observed clearly meets the definition of sexual behavior. We did not observe penile-anal intromission. However, the social function of sexual behavior is not strictly tied to penetration and does not always involve mounting (Dixson 2010). Non-reproductive sexual behavior, including all forms of SSB, can be an efficient way of social communication (Delval et al. 2023). Some aspects of the behavior, including the secrecy away from other group members without responding to calls, resonate with descriptions of different-sex sexual behavior in spider monkeys (Campbell and Gibson 2008).

Grappling can serve as the basis for sexual behavior (between individuals of the same or different sexes). However, since genital manipulation does not always occur, grappling is not necessarily sexual behavior (Eisenberg and Kuehn 1966; Schaffner et al. 2012). This complex social behavior should, therefore, be interpreted as a means of testing or strengthening bonds (especially between philopatric males) and regulating social uncertainty.

The observed case aligns with descriptions of grappling in other species of *Ateles* including Geoffroy’s (*A. geoffroyi*: Schaffner et al. 2012) and white-bellied spider monkeys (*A. belzebuth*: Eisenberg and Kuehn 1966). Since grappling consists of a series of specific behaviors related to courtship and is not accompanied by play vocalizations or play face, it can be clearly recognized as such and differentiated from a play bout. Previous observations of grappling mostly involved individuals of different age classes, and were typically initiated by a younger (juvenile or subadult) male toward an older (subadult or adult) male (Aureli and Schaffner 2008; Schaffner et al. 2012). In our case, both males were subadults. However, we are not fully aware of the social context of the group, and did not have information on the social position of the individuals, such as the exact age. Since we arrived when grappling had already been underway, we did not see which individual initiated the grappling bout, but both subadult males restarted the behavioral sequences after the interruptions when they sat on the branch.

Our report contributes valuable insights to the broader view of primate sexual behavior as the first documentation of SSB in brown-headed spider monkeys and the first documented case of SSB between subadult males. Furthermore, our observation highlights the risky nature of grappling, because it may involve the handling of an individual’s genitalia with a conspecific’ mouth or hands. The observation also fits within current sociosexual interpretations of SSB in animals as strengthening social relationships (Bagemihl 1999) and regulating tension (Clay and de Waal 2015). This broader interpretation also applies to non-reproductive sexual behavior between individuals of different sex.

## Data Availability

There is no data available associated to this article.
